# Melatonin Mediates Cardiac Tissue Damage under Septic Conditions Induced by Lipopolysaccharide

**DOI:** 10.3390/ijms252011088

**Published:** 2024-10-15

**Authors:** Milan Lazarević, Miloš Kostić, Tanja Džopalić, Danka Sokolović, Zorica Lazarević, Jelena Milovanović, Vanja Ničković, Dušan Sokolović

**Affiliations:** 1 Department of Immunology, Medical Faculty of Niš, University of Nis, 18000 Niš, Serbia; dr_m.lazarevic@hotmail.com (M.L.); milos.kostic@medfak.ni.ac.rs (M.K.); tanjche80@gmail.com (T.D.); 2 Clinic for Cardiovascular and Transplant Surgery, University Clinical Centre of Nis, 18000 Niš, Serbia; zoricalazarevic79@gmail.com; 3 Blood Transfusion Institute Niš, 18000 Niš, Serbia; dankasokolovic@gmail.com; 4 Faculty of Medicine, Unisversity of Priština, 38220 Kosovska Mitrovica, Serbia; jelena_krdzic@yahoo.com; 5 Clinic of Gastroenterohepatology, University Clinical Centre of Niš, 18000 Niš, Serbia; nickovicvanja@gmail.com; 6 Institute for Biochemistry, Faculty of Medicine, University of Niš, 18000 Niš, Serbia

**Keywords:** melatonin, lipopolysaccharide, heart, inflammation

## Abstract

Lipopolysaccharide (LPS) is known to induce oxidative stress and inflammation, leading to significant damage in cardiac tissues. This study investigates the protective effects of melatonin (MLT) against LPS-induced oxidative damage, inflammation, and apoptosis in rat heart tissue. Rats were divided into four groups (*n* = 6 per group): control, melatonin-treated, LPS-treated, and LPS + melatonin-treated. Oxidative stress markers, including thiobarbituric acid-reactive substances (TBARSs) and advanced oxidation protein products (AOPPs), were measured. Additionally, inflammatory markers, such as interleukin-6 (IL-6) levels, inducible nitric oxide synthase (iNOS) and nitric oxide (NO) content, and apoptotic markers, caspase-3, caspase-9, and acidic DNase activity, were evaluated. LPS treatment significantly increased TBARS, AOPP, and IL-6 levels, as well as the activity of caspase-3, acidic DNase and iNOS and NO content compared to the control group. Co-treatment with melatonin significantly reduced the levels of TBARS and AOPP levels, and caspase-3 and acidic DNase activities nearly matched those of the control group, while caspse-9 was still slightly increased. Interestingly, IL-6, iNOS and NO levels were significantly decreased but did not fully match the values in the control group. Melatonin mitigates LPS-induced oxidative stress, inflammation, and apoptosis in rat heart tissue by affecting all studied parameters, demonstrating its potential as a therapeutic agent for conditions characterized by oxidative stress and inflammation. Further research is warranted to explore the clinical applications of melatonin in cardiovascular diseases.

## 1. Introduction

Sepsis is characterized by multiple organ failure resulting from an intense inflammatory response of the host to infection [1], and the disease’s progression in some severe cases exhibits high mortality rates [2]. According to the official definition, sepsis is a life-threatening organ dysfunction caused by a dysregulated host response to infection. Severe sepsis can progress to septic shock, involving circulatory (hypotension) and metabolic dysfunction and an elevated risk of mortality [3], and is known to be associated with acute mitochondrial damage, which is further linked to the development of multi-organ failure [4]. Sepsis-induced myocardial disfunction can be associated with impaired cardiac muscle circulation, a direct damaging effect on myocardium or impaired mitochondrial function [4]. Mitochondrial damage could be explained through hypoperfusion and volume redistribution, followed by free oxygen radical (reactive oxygen species (ROS)) formation and autophagy [5]. During sepsis, the imbalance between proinflammatory and anti-inflammatory signals is also believed to result in tissue damage, vascular dysfunction, and multiple organ failure [6].

Lipopolysaccharide (LPS), an endotoxin from Gram-negative bacteria, is a crucial factor causing sepsis [7], which is known to be involved in sepsis and septic cardiomyopathy [8]. Cardiac muscle damage induced by LPS is mediated via the activation of Toll-like receptor 4 (TLR-4) [9,10], and further, the activation of a cascade of events leading to cardiac cell apoptosis is mediated via a calcium-sensing receptor [11]. Also, during sepsis, there is a notable disturbance in intracellular calcium homeostasis, followed by a dysfunction in the process of excitation/contraction coupling, which can be associated with cardiovascular complications such as systemic hypotension followed by the hypoperfusion of different organs [8]. The changes in the heart contractility associated with LPS have been found in various animal species and are attributed to altered loading conditions rather than to reduced contractility [12]. 

Melatonin (MLT) is a neurohormone synthesized from tryptophan and primarily produced by cells in the pineal gland [13,14,15,16]. While it is commonly known for its role in regulating circadian rhythms, MLT also exhibits different beneficial properties [17], such as antioxidant, anti-inflammatory, anti-apoptotic and immunostimulatory effects [16,17,18]. Previous studies have demonstrated MLT’s capacity to restore mitochondrial ATP production [19] by restoring mitochondrial electron transport chain function [20].

Despite our understanding, at least in part, of the pathophysiology of sepsis, there is still no adequate nor fully effective therapeutic option for its treatment. Thus, efforts to suppress sepsis development and cardiac damage induced by LPS should focus on inhibiting proinflammatory cytokines and molecules associated with the cell signaling cascade leading to apoptosis. Hence, in the present study, we aimed to examine how dose co-treatment with MLT impacts rat cardiac tissue damage associated with LPS endotoxemia. This was carried out by studying the changes in inflammatory and apoptotic signaling within the tissue, as well as oxidative tissue damage.

## 2. Results

The serum activities of lactate dehydrogenase (LDH) and creatine kinase (CK-MB) were found to be significantly increased in animals exposed to LPS compared to the control group (Table 1). The application of MLT prevented an increase in both of these parameters; however, the values were still increased but were significantly different from the LPS-treated animals (Table 1).

Heart tissue TBARS and AOPP content was found to be significantly increased in rats treated with LPS (Table 2) compared to the control group. The application of MLT together with LPS led to a partial decrease in TBARS and AOPP when compared to LPS-treated rats (Table 2), with the values being almost identical to the ones in the control group since there was no significant difference between them.

**Figure 1:**
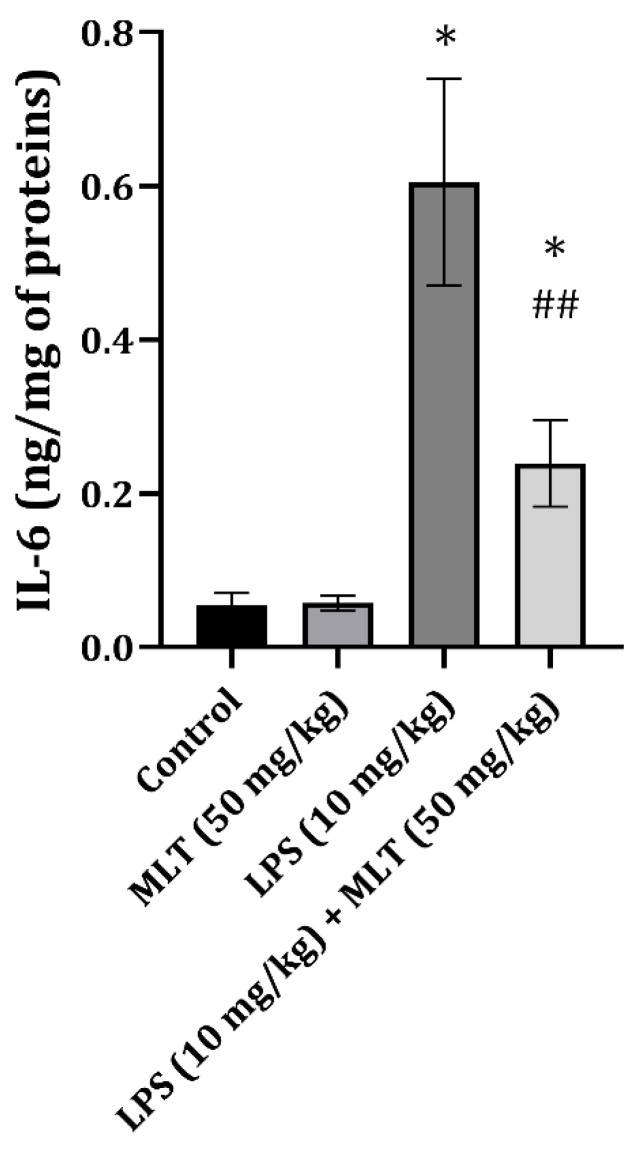
The effects of MLT on LPS-induced change in cardiac tissue IL-6 content (ng/mg of protein). The data are presented as mean ± S.D. (*n* = 6). The comparison was conducted using one-way ANOVA followed by Tuckey’s post hoc test, * *p* < 0.0001 vs. control, ^##^ *p* < 0.001 vs. LPS-treated animals.

The acute exposure of rats to LPS leads to a significant increase in cardiac tissue IL-6 content. Co-treatment with MLT (50 mg/kg) caused a significant decrease in IL-6. However, the values were still higher than those for the control group (Figure 1).

**Figure 2:**
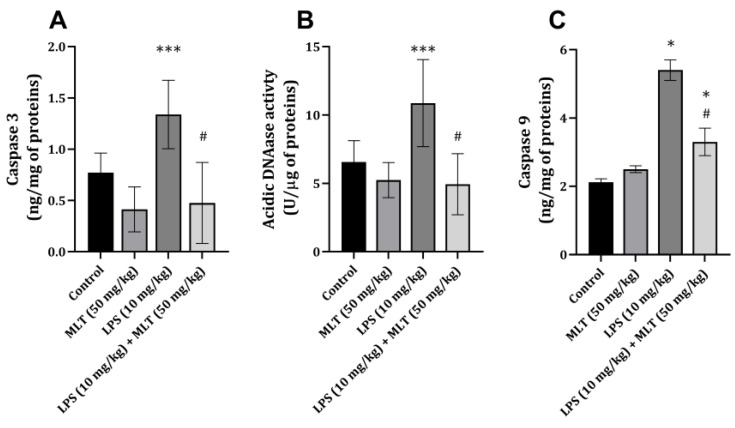
Effect of MLT on LPS-induced changes in heart tissue (**A**) caspase 3 (ng/mg of proteins), (**B**) acidic DNase (U/μg of proteins) activity and (**C**) caspase-9 (ng/mg of proteins). The data are presented as mean ± S.D. (*n* = 6). Comparison was carried out using one-way ANOVA followed by Tuckey’s post hoc test, * *p* < 0.001, *** *p* < 0.05 vs. control, ^#^ *p* < 0.001, vs. LPS-treated animals.

The administration of LPS led to a significant increase (*p* < 0.05) in caspase-3 and caspase-9 content and acidic DNase activity in rat heart tissue (Figure 2A,B). When the MLT was applied together with LPS, a significant diminution in the three studied parameters, reflecting the apoptosis process, was found. The activity of the two enzymes was almost identical to that in the control groups (Figure 2A,B), while caspase-9 was still higher than that in the control group (Figure 2C).

**Figure 3:**
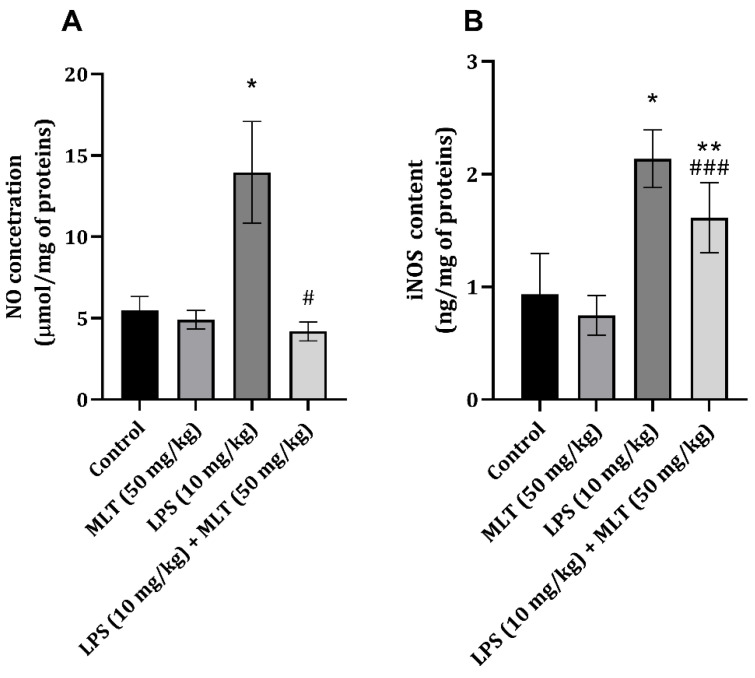
Effect of MLT on LPS-induced changes in heart tissue (**A**) nitric oxide (μmol/mg of proteins) and (**B**) iNOS (ng/mg of proteins) concentrations. The data are presented as mean ± S.D. (*n* = 6). Comparison was carried out using one-way ANOVA followed by Tuckey’s post hoc test, * *p* < 0.001, ** *p* < 0.01 vs. control, ^#^ *p* < 0.001, ^###^ *p* < 0.05 vs. LPS-treated animals.

LPS application increased iNOS and NO content in the cardiac tissue of exposed rats. Furthermore, MLT application, together with LPS, decreased the two studied parameters (Figure 3A,B). The decrease in NO was more pronounced than in the content of iNOS, which reflected the significance of the decrease compared to the control of the studied parameters.

## 3. Discussion

The binding of LPS to corresponding receptors triggers cascading signaling pathways, leading to an overwhelming release and production of proinflammatory cytokines [6]. Also, LPS causes an increase in ROS production [5], which further damages cell-building molecules such as lipids and proteins. In the present study, LPS caused a significant increase in TBARS and AOPP content in heart tissue (Table 2). The damage of tissue macromolecules associated with LPS application could have provoked the release of the intracellular enzymes such as LDH and CK-MB (Table 2). ROS-damaged lipids and proteins can activate various signaling pathways, including MAPK, JAK/STAT, and NF-κB [21], which further leads to the production of proinflammatory cytokines like IL-6 and the expression of iNOS, increasing NO production [22]. Apart from the damage to muscle cells, ROS damage to endothelial cells results in the upregulation of adhesion molecules, promoting leukocyte adhesion and transmigration, further enhancing the inflammatory response, with NO playing a dual role in modulating vascular tone and inflammation [23].

Melatonin interacts with ROS through multiple mechanisms to prevent oxidative damage to lipids and proteins. One of them involves direct radical scavenging ability [24], the inhibition of ROS generation and busting antioxidant enzyme production [25]. By scavenging ROS, enhancing antioxidant defenses, and stabilizing cellular structures, melatonin effectively inhibits the formation of TBARS and AOPP (Table 1) and the release of LDH and CK-MB (Table 2). This activity of MLT can come from an improvement in mitochondrial function and reduction in mitochondrial ROS production, thus limiting one of the greatest sources of cellular oxidative stress [19,20].

Interleukin 6 is a proinflammatory cytokine produced during infections and is a well-known predictor of blood culture positivity in patients with sepsis [26]. Apart from this function, IL-6 causes cardiac muscle dysfunction through the sirtuin 1 pathway [27] and promotes cardiac hypertrophy through the activation of fgp130 [28]. Interestingly, some studies suggest that an acute increase in IL-6 causes cardio-protection in the early stages of sepsis, decreases the process of apoptosis, and prevents oxidative damage [29]. In the present study, a significant increase in IL-6 was found in cardiac tissue (Figure 1), and the co-treatment with MLT partially ameliorated this increase. This partial decrease might be a reflection of the protective potential of IL-6, which reflected the apoptosis process (Figure 2) and balanced the oxidative damage (Table 2).

Caspase-3, caspase-9 and DNase II play complementary roles in the process of apoptosis. Caspase-3 is primarily responsible for the activation of nucleases that initiate DNA fragmentation, while DNase II ensures the complete degradation of DNA within the acidic environment of lysosomes during the clearance of apoptotic cells [30]. Caspase-9 is associated with mitochondria-mediated apoptosis, while mitochondria are recognized for their importance in cardiac disfunction under septic conditions [4], including ones seen after LPS application [11]. This coordinated activity is essential for the orderly and efficient removal of dying and/or damaged cells, in that way maintaining tissue homeostasis. In the present case, an increase in all mentioned enzyme activities was observed (Figure 2A–C), suggesting that apoptosis is occurring to a certain extent in the cardiac tissue following LPS application. Also, the mentioned connection between IL-6 and apoptosis has been shown in certain cancer cell lines, where IL-6 increases caspase-3 activation [31], thus further activating the progression of programmed cell death.

Melatonin was also found to prevent apoptosis through different processes, which might involve the modulation of oxidative damage or the direct inhibition of a signaling process [17,21]. Apart from oxidative damage prevention, MLT results in the activation of Poly (ADP-ribose) polymerase (PARP) and Bcl proteins, preventing the apoptosis process [32]. However, a single path of the anti-apoptosis action of MLT cannot be pinpointed but should instead be observed through a panel of molecular signals that mutually interact. Interestingly, MLT did not fully prevent the observed increase in caspase-9, which followed LPS application, suggesting that there is some mitochondrial damage present in the process.

The application of LPS led only to a slight, although significant, increase in heart tissue iNOS and NO content, which is, to a certain extent, expected since cardiac muscle tissue is mainly composed of muscle cells. Melatonin was previously proven to act through the iNOS/NO pathway and thus prevent heart tissue damage induced by CCl_4_ [33], and in murine macrophage culture, it was proven to decrease iNOS mRNA content [34]. By inhibiting iNOS and consequently NO, an easier and more prompt remodeling of the damaged heart can occur, and this could, in the end, prevent myocardial dysfunction and improve cardiac reserve [35]. Thus, based on the results of the present study concerning iNOS and NO content, at least part of the activity of MLT could be attributed to the direct inhibition of NO synthesis, as has also been previously proven [36]. Also, another part of the decrease in NO content can be through a direct interaction between MLT and NO since these two share various cellular compartments [37], which could further explain the incomplete decrease in iNOS content observed in the group of animals treated with LPS and MLT (Figure 3B).

## 4. Materials and Methods

### 4.1. Drugs and Chemicals

Lipopolysaccharide (LPS) from *Escherichia coli* O111:B4 and MLT were procured from Sigma (St. Louis, MO, USA) and administered intraperitoneally (i.p.) to the animals. For the induction of septic shock, a single dose of LPS at 10 mg/kg was administered. MLT solutions were prepared at a dose of 50 mg/kg before application [16]. Ketamine, purchased from Richter Pharma AG (Wels, Austria), served as a general anesthetic. All other chemicals used were of analytical-grade purity.

### 4.2. Animals and Housing

Healthy male Wistar albino rats (7 weeks old, weighing 150 to 200 g) were obtained from the Vivarium of the Institute of Biomedical Research, the Faculty of Medicine, the University of Niš, Serbia. The rats were housed under standard laboratory conditions, maintaining a temperature of 22 ± 2 °C, a relative humidity of 50 ± 5%, and a 12/12 h light/dark cycle. They had ad libitum access to standard commercial laboratory food and water. The study received approval from the local Ethics Committee, and all experimental procedures adhered to ethical regulations, including the Helsinki and European Community guidelines for the ethical handling of laboratory animals (the E.U. Directive of 2010; 2010/63/E.U.) and the Guide for the Care and Use of Laboratory Animals (8th edition, National Academies Press), as well as the laws of the Republic of Serbia.

### 4.3. Experimental Design

Rats were randomly assigned to four groups, each comprising 6 rats. The treatment schedule at the start of the experiment, previously established [38], was as follows:

Group I: Vehicle—0.8% ethanol in saline at a dose of 10 mL/kg.

Group II: MLT—a single 50 mg/kg dose of MLT administered by oral gavage.

Group III: LPS—a single i.p. injection of LPS at a dose of 10 mg/kg.

Group IV: LPS + MLT—a single 50 mg/kg dose of MLT followed by a single 10 mg/kg dose of LPS.

Twelve hours post treatment, the animals were euthanized with an overdose of ketamine, and heart tissues were collected from the animals for further analysis.

### 4.4. Tissue Isolation and Homogenate Preparation

Whole heart tissue specimens were finely cut, homogenized (10% *w*/*v*) in ice-cold phosphate-buffered saline, and centrifuged (12,000 rpm for 10 min at 4 °C) to obtain clear supernatants. The protein content in the supernatants was measured using Lowry’s method [39] with a standard bovine serum albumin curve.

### 4.5. Oxidative Tissue Damage Determination

Thiobarbituric acid reactive substances (TBARSs), indicative of lipid peroxidation in the liver, were quantified spectrophotometrically. This involved a reaction between thiobarbituric acid and modified lipids, following the method described by Stojanović et al. [40]. The absorbance of the resulting colored product was measured at 532 nm, and the outcomes were presented as nanomoles of TBARS per milligram of tissue proteins.

Advanced oxidation protein product (AOPP) determination in the heart samples was conducted through the spectrophotometric detection method previously described [41]. The assay operates on the principle of a colorimetric reaction between proteins and chlorinated oxidants. Initially, the samples were diluted at a ratio of 1:10 in phosphate-buffered saline (PBS), and subsequently, 15 μL of potassium iodide was added to each well of a microtiter plate. Afterward, 30 µL of glacial acetic acid was added to all wells. The absorbance was measured at 340 nm, and the absorbance of the sample blank was subtracted from the sample wells. The calculated chloramine-T equivalent concentrations of AOPPs were normalized to the total protein of each patient and expressed in µM/mg protein.

### 4.6. Inflammatory Cytokine Determination

The amount of IL-6 was quantified using a standard ELISA kit (Quantikine ELISA Rat IL-6, R&D Systems, Minneapolis, MN, USA; R6000B), following the manufacturer’s instructions. 

### 4.7. Apoptosis Estimation

To estimate the process of apoptosis, acidic DNAase (DNase II (E.C. 3.1.22.1)) was determined in heart tissue homogenate. Methods for measuring acidic DNAase activity included using DNA as a substrate, while the reaction was carried out under optimum pH = 5.0 and using Mg^2+^ ions as activators [42]. The activity of the enzyme was expressed as U/μg of proteins.

The activity of caspase-3 in heart tissue homogenates was measured by a colorimetric assay (ab39401; Abcam Inc., Cambridge, MA, USA) following the manufacturer’s instructions. The obtained data are presented as ng/mg of proteins.

The content of caspase-9 in heart tissue homogenates was measured by a sandwich ELISA assay (E-EL-R0163; Elabscience, USA), following the manufacturer’s instructions. The obtained data are presented as ng/mg of proteins.

### 4.8. Nitric Oxide and iNOS Determination

After the deproteinization of heart tissue, the production of nitric oxide (NO) was assessed by measuring the concentrations of nitrite and nitrate (NO^2−^ and NO^3−^, respectively). The absorbance of nitrates was determined spectrophotometrically using Griess reagent [43]. The results were expressed in nmol/mg of heart tissue protein.

The iNOS content was estimated using an ELISA (CUSBIO, CSB-E08325r, Houston, TX, USA) sandwich enzyme immunoassay kit, following the manufacturer’s instructions. The obtained values are expressed as I.U./mg of tissue proteins.

### 4.9. Statistical Analysis

The data obtained were expressed as mean values ± standard deviation (S.D.). Statistically significant differences were assessed using a one-way analysis of variance (ANOVA), followed by Tukey’s post hoc test for multiple comparisons (conducted with GraphPad Prism version 5.03, San Diego, CA, USA). Probability values (*p*) equal to or less than 0.05 were considered statistically significant.

## 5. Conclusions

This study showed that there are extensive changes in the rat heart tissue during endotoxemia induced by lipopolysaccharide injection, which reflects the tissue oxidative status, IL-6 concentration, apoptosis process, and NO signaling. The administration of melatonin, a neurohormone, together with lipopolysaccharide, mitigated the changes in heart tissue, most probably through the prevention of the oxidative damage of lipids and proteins. Also, the prevention of IL-6 content increase and NO signaling resulted in decreasing the apoptosis process in the heart tissue. These actions highlight the potential of melatonin as a therapeutic agent in conditions associated with oxidative stress and inflammation. Once again, melatonin has proven to possess beneficial properties in different tissue damage models, pointing out that it might possess clinical usage, which needs to be studied.

## Figures and Tables

**Table 1:** Serum parameters for LDH and CK-MB in rats of different experimental groups.

Group/Parameter	LDH (U/L)	CK-MB (U/L)
Control	537 ± 85	1.09 ± 0.14
Melatonin	614 ± 94	1.23 ± 0.07
LPS	1588 ± 197 *	1.90 ± 0.20 *
LPS + Melatonin	1172 ± 70 ^#^	1.04 ± 0.27 ^#^

The data are presented as mean ± S.D. (*n* = 6). The comparison was carried out using one-way ANOVA followed by Tuckey’s post hoc test, * *p* < 0.0001 vs. control, ^#^ *p* < 0.0001 vs. LPS-treated animals.

**Table 2:** Heart tissue oxidative parameters in rats belonging to different experimental groups.

Group/Parameter	TBARS (nM/mg of Protein)	AOPP (µM/mg of Protein)
Control	1.97 ± 0.33	1.09 ± 0.14
Melatonin	2.29 ± 0.35	1.23 ± 0.07
LPS	5.25 ± 1.24 *	1.90 ± 0.20 *
LPS + Melatonin	2.49 ± 0.3 ^#^	1.04 ± 0.27 ^#^

The data are presented as mean ± S.D. (*n* = 6). The comparison was conducted using one-way ANOVA followed by Tuckey’s post hoc test, * *p* < 0.0001 vs. control, ^#^ *p* < 0.0001 vs. LPS-treated animals.

## Data Availability

The data are available upon reasonable request from the corresponding author.
